# A Study Examining the Impact of County-Level Demographic, Socioeconomic, and Political Affiliation Characteristics on COVID-19 Vaccination Patterns in Indiana

**DOI:** 10.3390/ijerph21070892

**Published:** 2024-07-09

**Authors:** Giuseppe Pangan, Victoria Woodard

**Affiliations:** Department of Applied & Computational Mathematics & Statistics, University of Notre Dame, Notre Dame, IN 46556, USA; vweber1@nd.edu

**Keywords:** COVID-19 vaccinations, demographics, geographic health information systems, vaccine uptake, counties

## Abstract

The COVID-19 vaccination campaign resulted in uneven vaccine uptake throughout the United States, particularly in rural areas, areas with socially and economically disadvantaged groups, and populations that exhibited vaccine hesitancy behaviors. This study examines how county-level sociodemographic and political affiliation characteristics differentially affected patterns of COVID-19 vaccinations in the state of Indiana every month in 2021. We linked county-level demographics from the 2016–2020 American Community Survey Five-Year Estimates and the Indiana Elections Results Database with county-level COVID-19 vaccination counts from the Indiana State Department of Health. We then created twelve monthly linear regression models to assess which variables were consistently being selected, based on the Akaike Information Criterion (AIC) and adjusted R-squared values. The vaccination models showed a positive association with proportions of Bachelor’s degree-holding residents, of 40–59 year-old residents, proportions of Democratic-voting residents, and a negative association with uninsured and unemployed residents, persons living below the poverty line, residents without access to the Internet, and persons of Other Race. Overall, after April, the variables selected were consistent, with the model’s high adjusted R^2^ values for COVID-19 cumulative vaccinations demonstrating that the county sociodemographic and political affiliation characteristics can explain most of the variation in vaccinations. Linking county-level sociodemographic and political affiliation characteristics with Indiana’s COVID-19 vaccinations revealed inherent inequalities in vaccine coverage among different sociodemographic groups. Increased vaccine uptake could be improved in the future through targeted messaging, which provides culturally relevant advertising campaigns for groups less likely to receive a vaccine, and increasing access to vaccines for rural, under-resourced, and underserved populations.

## 1. Introduction

The COVID-19 pandemic greatly affected millions of lives in the United States and around the world, with the World Health Organization reporting over 774 million cases and over 7 million deaths worldwide [1]. The development of vaccines to reduce the effects and deaths associated with COVID-19 was expedited, and by February 2021 the U.S. Food and Drug Association issued emergency-use authorizations for three vaccines: Ad26.COV2.S (Johnson & Johnson-Janssen [New Brunswick, NJ, USA & Beerse, Belgium]), mRNA-1273 (Moderna [Cambridge, MA, USA]), and BNT162b2 (Pfizer-BioNTech [New York City, NY, USA & Mainz, Germany]) [2]. Although the vaccines were widely available and were demonstrated to be safe and effective for preventing adverse responses to COVID-19, the uptake across the United States from county to county was not uniform [3,4].

Communities with lower COVID-19 vaccination coverage included populations that had less access to the vaccine, including rural populations [5,6,7], economically disadvantaged neighborhoods, and populations that were unemployed or uninsured [6,8,9,10]. Other studies have written about how public health disasters like the COVID-19 pandemic and its subsequent vaccine rollout exacerbated already-existing disparities within and between communities [11,12,13]. For example, other studies have noted that the BIPOC (Black, Indigenous, and Other People of Color) population in the United States had an increased risk of poorer outcomes related to COVID-19 because of underlying health conditions within the population, including diabetes, hypertension, and cardiovascular disease [14]. Furthermore, there is an inherent mistrust in the medical field in the BIPOC community that exists because of a long history of health policies that have negatively affected these populations, and this skepticism has resulted in the undertesting and undervaccination of these medically vulnerable populations during the pandemic [14,15]. Furthermore, public health disasters negatively affect disadvantaged socioeconomic groups through losses of housing or employment, disruption to systems of education, and increased rates of food security and poverty [16,17,18,19]. Other studies explained how zip codes with the highest vaccination coverage had more access to the vaccine and had lower Social Vulnerability Index scores, with the contrapositive also being true [8,20,21]. The Social Vulnerability Index is a measure created by the Centers for Disease Control and Prevention and Agency for Toxic Substances and Disease Registry that measures and quantifies communities that experience social vulnerability, and the demographic and socioeconomic characteristics that can negatively impact a community’s response to public health disasters like pandemics or natural disasters [22].

At the same time, other communities with lower COVID-19 vaccination coverage included populations that were hesitant to receive the vaccine because of political ideologies and those who have mistrust in public health officials [23,24]. In particular, socially and economically disadvantaged groups who have a distrust in the government are less likely to heed messaging about vaccination [25]. In addition, there are those populations that had ample access to the vaccine, but the vaccination attitudes and mixed messages shown by Republican leadership slowed down vaccination trends [26,27,28,29,30].

Studies have leveraged geographic health information systems to study not only the local and global effects of COVID-19 cases and vaccinations, but also broader public health research [12,31,32,33]. These methods have used geographic building blocks, like the county-level or the census tract-level, to describe social and economic disparities among differing populations in their access to COVID-19 testing [34,35,36,37] and vaccinations [26,38].

The objective of this study was to determine what demographic, socioeconomic, and political affiliation variables affected the patterns of COVID-19 vaccinations for a single midwestern state over the first 12 months of vaccine rollout. This objective holds three specific distinctions. The first is that we wished to see how the three categories of demographic, socioeconomic, and political variables interacted to affect vaccination rates, and not just as independent variables. Second, a single state, Indiana, was chosen to determine if the aggregated findings of national-level studies are reasonable when looking at more localized regions. Finally, we wanted to see if there was consistency over time, or if changes occurred in vaccine uptake over time, due to different variables. We constructed a dataset that linked the vaccination data with the demographic, socioeconomic, and voter data for Indiana’s 92 counties. From there, we constructed monthly linear regression models to determine the effects of certain county characteristics on the county’s COVID-19 vaccination patterns.

## 2. Materials and Methods

### 2.1. Data Collection

The study area consisted of all 92 counties in Indiana. As part of a larger study, we obtained daily county-level aggregates of COVID-19 vaccinations from the Indiana State Department of Health [39] between 26 February 2020 and 7 July 2022 (n = 79,396 county-days). For the purposes of this study, we excluded observations before 1 January 2021 (n = 28,520 county-days) because COVID-19 vaccinations were not widely available to the public. Additionally, we excluded observations after 31 December 2021 (n = 17,296 county-days) due to a sharp drop off in first vaccination rates after this date and because, at this point, anyone who wanted to receive the vaccine could do so. The final dataset comprised 33,580 county-day observations. To fit the methodology, and to more tangibly explore spatial and temporal trends, the data were summarized by month, comprising 2760 county–month observations.

We obtained demographic data from the 2016–2020 American Community Survey Five-Year Estimates [40]. This included data from several tables, including Age and Sex [S0101], Educational Attainment [S1501], Income in the Last Twelve Months [S1901], Demographic and Housing Estimates [DP05], Population Under 18 Years of Age [B09001], Language Spoken at Home [S1601], Health Insurance [S2701], Internet Access [S2801], and Percent Disabled [S1810]. We also obtained political affiliation data from the Indiana Elections Results Database for the 2020 Presidential Election [41]. We linked these data to COVID-19 vaccination counts at the county level.

### 2.2. Variables of Interest

**Figure 1:**
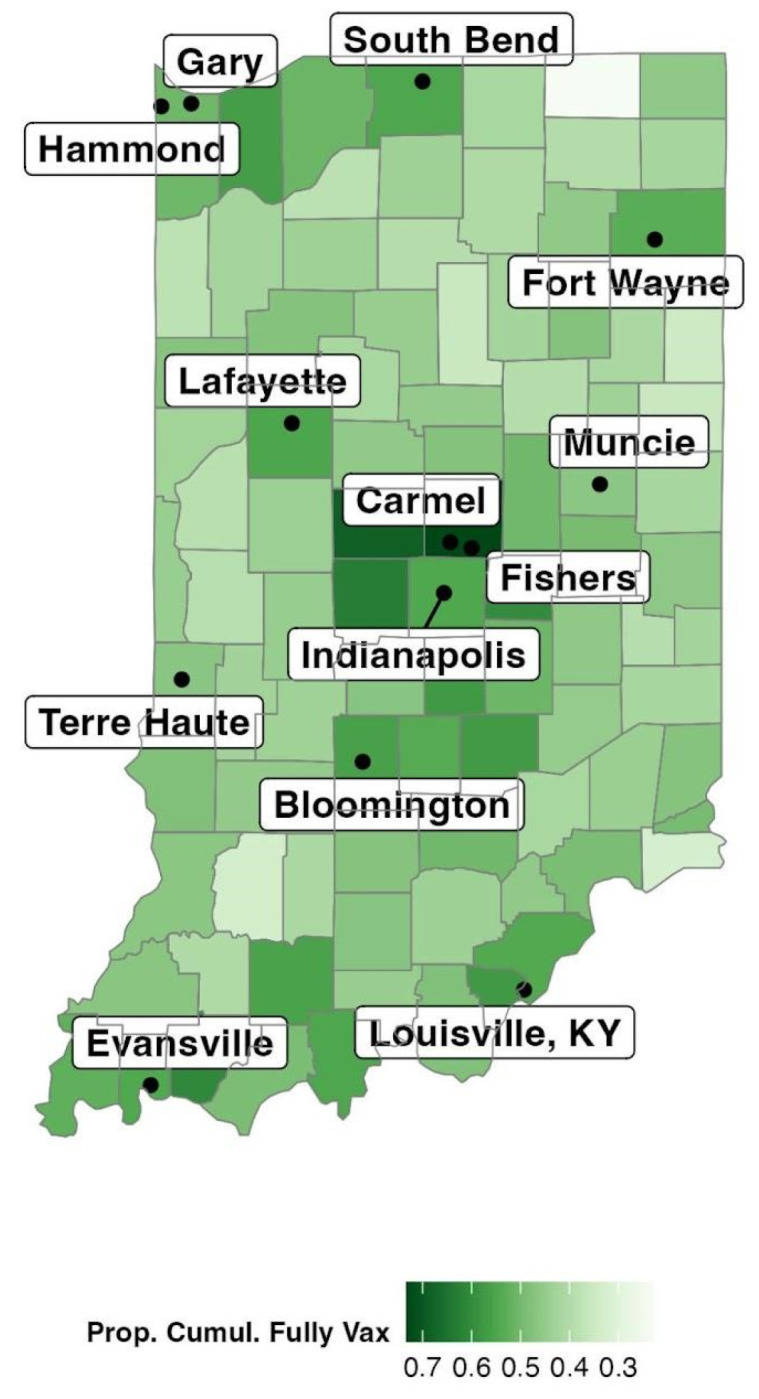
Proportion of COVID-19 Fully Vaccinated Individuals in Indiana. Data Source: [39].

The outcome of interest was COVID-19 fully vaccinated persons per 100,000 people. We adopted the definition of a COVID-19 fully vaccinated individual as someone who received either two doses of mRNA-1273, two doses of BNT162b2, or one dose of AD26.COV2.S as compiled by the Indiana State Department of Health. Figure 1 maps out the proportion of the population that was fully vaccinated at the end of 2021.

Consistent with previous studies examining disparities due to county-level demographic, socioeconomic, and political characteristics, the independent variables we wished to include in the model (either implicitly or explicitly) were county-level financial resources, educational attainment, and political affiliation [28,41]. Previous studies have also reported differences in COVID-19 exposure, vaccine uptake, and access to healthcare across various demographics and geographic units [10,42,43,44]. To account for this in our own modeling, we chose to include covariates for age, race and ethnicity, uninsured populations, and rurality.

#### 2.2.1. Demographic Variables

For age, we constructed four groups based on the proportion of residents <18 years-old, 18–39 years-old, 40–59 years-old, and 60+ years-old. For race and ethnicity, since most counties were racially homogenous and predominantly composed of non-Hispanic White residents, we created two variables: proportion of non-Hispanic White residents and proportion of residents of other race (including Hispanic, Asian, Native Hawaiian/Pacific Islander, Black, and American Indian/Alaska Native). We also obtained county proportions of non-English speakers, gender, and persons with disabilities [20] from the 2016–2020 American Community Survey Five-Year Estimates [40,45].

#### 2.2.2. Socioeconomic and Political Variables

To operationalize financial resources, we obtained median household income, unemployment rates, uninsured rates, and county population below poverty from the 2016–2020 American Community Survey Five-Year Estimates [37]. To operationalize educational attainment, we calculated the proportion of 18+ year-old residents with Bachelor’s degrees, using the number of persons with a Bachelor’s degree for both the 18–24-year-old and the 25 and over age groups, divided by their population sizes separately, to get the appropriate proportions. To operationalize political affiliation, the proportion of Democratic-voting residents, Republican-voting residents, and Other Party-voting residents in the 2020 U.S. Presidential Election was calculated with data from the Indiana Elections Results Database. From this database, we extracted the counts for individuals who voted for the Democratic, Republican, and Other parties by county, then divided those counts by the county’s population, found in the 2020 American Community Survey.

#### 2.2.3. Geographic Variables

**Figure 2:**
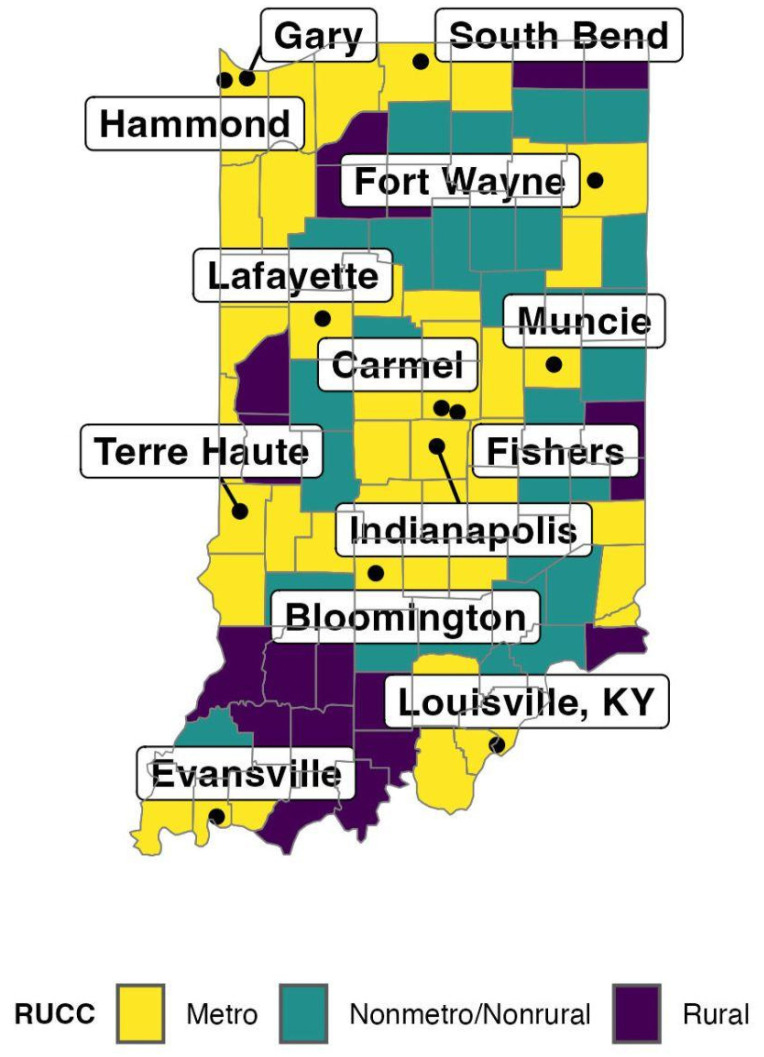
Indiana Rurality Map. Data Source: [46].

For rurality, we utilized the county classifications by the 2020 U.S. Department of Agriculture Rural–Urban Commuting Codes (RUCC) [46]. This set of codes, based on the 2020 U.S. Census, assigns a county a value of 1–9, which indicates the urbanity or rurality of that county. This is based on characteristics such as commuting distance and population density. Table 1 includes the description of the nine levels of the Rural–Urban Commuting Codes. Some of these identifications included only one or two counties in the state, which did not provide enough variation to be useful in modeling. To aid in the modeling process, the nine categories were regrouped as metro, non-metro/non-rural, and rural, which was dependent on both the largest urban population within the county and the adjacency of the county to a metro area. The designations we used in our analysis are also provided in Table 1. Figure 2 is a map that indicates our classifications for rurality for each Indiana county. The rurality was included in the analysis because we wanted to model population density, and therefore overall differences in COVID-19 exposure and access to healthcare and vaccination sites [47].

#### 2.2.4. Summary of the Variables

A summary of these variables, including the average value, the standard deviation, and the maximum and minimum values across all 12 months and all 92 counties can be found in Table 2. This table demonstrates that, for some of the collected variables in Indiana, such as the age categories, unemployment, poverty rate, and race, the standard deviation is small, indicating that the variable is mostly homogeneous throughout Indiana. At the same time, for variables such as the proportion of college-educated individuals and Republican voters, the standard deviation is high, demonstrating that the variable is heterogeneous and has a wide range of values.

**Figure 3:**
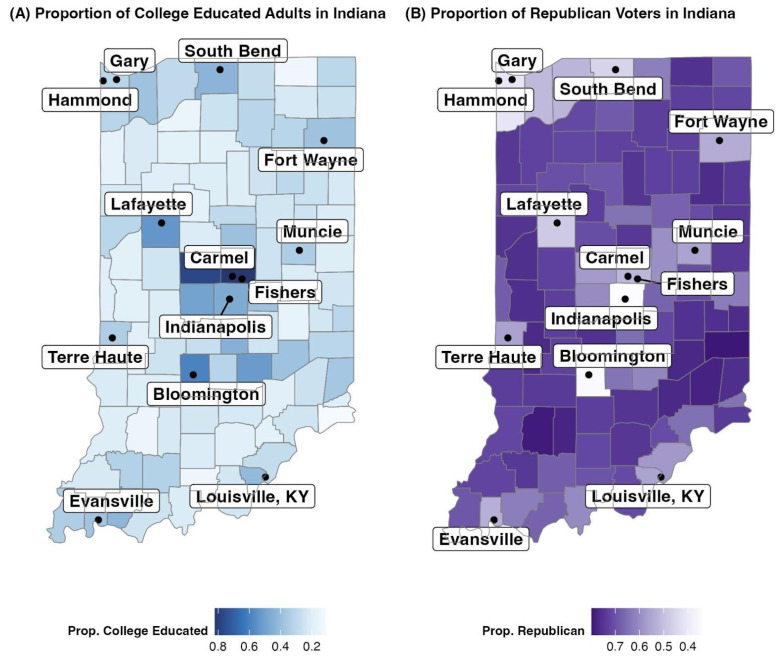
(**A**) Proportion of College-educated Adults in Indiana. Data Source: [41]. (**B**) Proportion of Republican voters in Indiana. Data Source: [42].

**Figure 4:**
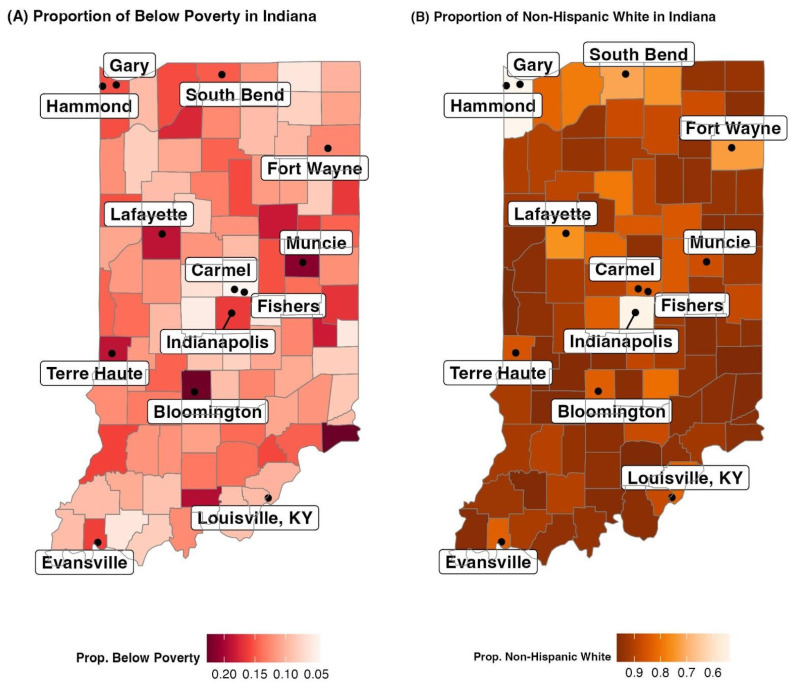
(**A**) Proportion of Population Below Poverty in Indiana. Data Source: [40] (**B**) Proportion of Non-Hispanic White Population in Indiana. Data Source: [40].

The homogeneity and heterogeneity of the variables in Indiana are further seen in Figure 3 and Figure 4. In Figure 3A, the map shows that the counties with the highest proportion of Bachelor’s degree-holders are mainly concentrated around cities like Indianapolis and its suburbs Gary and Fort Wayne, as well as college towns like West Lafayette (Purdue University), Bloomington (Indiana University), and South Bend (University of Notre Dame). In Figure 3B, this map demonstrates that non-metro and rural counties are more likely to have higher proportions of Republican voters, while the inverse is also true—counties with major population centers and cities are less likely to lean Republican.

In Figure 4A, the map shows that counties that are home to major cities and population centers are more likely to have higher proportions of the population below poverty than non-metro and rural counties. In Figure 4B, the map demonstrates that the state of Indiana is mostly homogeneous with regards to race, with most counties having 80% or more of its population as Non-Hispanic White.

After synthesizing all the variables into a collective dataset, we analyzed the correlation between each of the variables to determine if there would be any strong multicollinearity between predictor variables. Through this analysis, the median household income variable was (not surprisingly) found to be moderately correlated with the proportion of the county population below poverty and the proportion of unemployed persons. After further analysis, median household income was excluded from the list of variables to be included in the model, in favor of poverty and unemployment. This was done for two reasons. First, the median household income was generalized over county level, so it did not capture the extreme maximum and minimum within a county. Whereas the proportions calculated for both poverty and unemployment levels include information from all residents of a county. Second, our analysis sought to identify variables that were important in predicting COVID-19 vaccination patterns. When median household income was included in the model, any variable that was somewhat correlated with it was removed from the model when conducting appropriate model-selection methods. That is, median household income tended to dominate the model over any other financial factors and did not allow us to see any of the other nuances of the financial aspects of a county that would affect our response variable.

### 2.3. Methodology

This section will focus on the creation of our model. Recall that our goal was to estimate the effects of county-level demographic and political affiliation characteristics on cumulative vaccinations per 100,000 persons for the study period.

As noted above, the daily vaccination data were first aggregated into monthly data. In particular, we divided the 2021 dataset into twelve months and created a model for each month of the form:

$$vaccine=\beta_{0}+\beta_{1\;}*\%Male+\beta_{2}*\%Below\;Poverty+\;\beta_{3}*\%College\;Educated+$$

$$\beta_{4}*\%Republican+\beta_{5\;}*\%Democratic+\beta_{6}*\%Other\;Party+$$

$$\beta_{7}*\%Non\;Hispanic\;White+\beta_{8\;}*\%OtherRace+\;\beta_{9}*\%Non\;English+$$

$$\beta_{10}*\%Age(18\;to\;39)+\beta_{11\;}*\%Age(40\;to\;59)+{\%\beta }_{12}*Age(60\;and\;up)+$$

$$\beta_{13}*RUCC(Nonmetro/Nonrural)+\beta_{14}*RUCC(Rural)+$$

$${\beta_{15}*\%\;Unemployment\;+\beta }_{16}*\%Uninsured+$$

$$\beta_{17}*\%Non\;Internet+\beta_{18}*\%Disability$$

The reader may note that the Metro RUCC category and the Age under 18 category are missing from the model. This is due to the categorical nature of the variables and how they were presented in the data set. When conducting the analysis, these categories get swept up in the intercept term and help serve as a baseline for their respective factor variable.

From this, one of our goals was to determine which variables were consistently left in the model from month to month after variable selection was conducted. While a more formal time series analysis, such as using the time lagged vaccine values, may have been a more appropriate type of analysis for this data, we decided not to utilize this approach. The reason for this was because the vaccine count per 100,000 from the previous time point was on a much larger scale than the other predictor variables in our model (most of which took on values of less than 1) and this variable overpowered the model, leaving little room for interpretation of the variables that were left in the model after variable selection.

For each individual monthly model, we evaluated its performance by selecting the most useful variables through the Akaike Information Criterion (AIC). Because the structure of the data was by county, and therefore potentially spatially correlated, we used an aerial spatial model with directly adjacent counties, in conjunction with the Moran test to check whether a formal spatial analysis was needed [29].

## 3. Results

In the models measuring vaccination patterns, those created for the months of January, February, and March did not appear to be very useful at predicting the number of vaccines administered, with none having an adjusted r-squared value higher than 52%. We believe this is because the vaccine had not been fully released in Indiana until the end of March (Table 3). However, from May through to the end of the year, almost all the variables in the AIC-selected model were the same. Additionally, none of these models produced significant results in the Moran test, suggesting that the spatial correlation between counties either did not exist or was already well modeled by the predictor variables used within the model. For completeness, the SAR and CAR models were also created for each month. However, the AIC values for both the SAR and CAR models were larger than the original linear model in all twelve months. Because of this, we continued to use the linear regression model for the remainder of the study. Table 3 provides more information on: the variables that were selected for each monthly model, including the slope coefficients for each of the variables, their standard error, and their *p*-values, as well as the adjusted r-squared for the model, the *p*-value for the Moran test, the AIC for the linear model, and the SAR and CAR models.

According to our aggregated model, there was a negative association found between cumulative vaccinations per 100,000 persons and the predictor variables of poverty rate, unemployment, uninsured, non-Internet, and proportion of the population that is of Other Race. A negative association indicated that the higher the value for the predictor variable, the lower the value for the vaccine rate, assuming all other variables in the model are held constant.

From the aggregated model we also found that there was a positive correlation between cumulative vaccinations per 100,000 persons and the predictor variables of ages 40–59, college-educated populations, and proportion of county Democratic voters. Table 3 summarizes the significant findings across all the models and variables.

**Figure 5:**
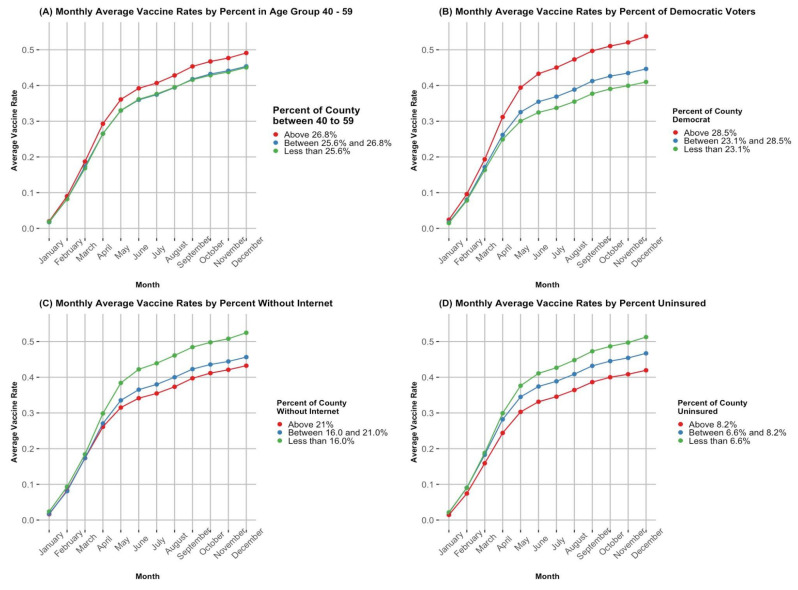
(**A**) A time series graph displaying the monthly average vaccination rate per month across counties in the lower, middle, and upper thirds of the county population proportion of ages 40–59. Data Source: [40] (**B**) A time series graph displaying the monthly average vaccination rate per month across counties in the lower, middle, and upper thirds of the county proportion of Democratic voters. Data Source: [41] (**C**) A time series graph displaying the monthly average vaccination rate per month across counties in the lower, middle, and upper thirds of the county proportion of the population without Internet access. Data Source: [40] (**D**) A time series graph displaying the monthly average vaccination rate per month across counties in the lower, middle, and upper thirds of the county proportion of uninsured individuals. Data Source: [40].

The graphs in Figure 5 demonstrate a selection of the relationships that we have discussed thus far. Each of these graphs shows the average vaccination rate across the 12 months in 2021, separated by levels of a predictor variable in the model. Each of these predictor variables was split into three categories, which represent the lower, middle, and upper thirds of the data for that variable. All counties that fell into that level of the predictor were used to find the average vaccination rate at the given time period. In the cases of ages 40–59 and the proportion of Democratic voters, we see the positive relationship between these variables and their impact on vaccine rate, with the counties that have the highest levels of the predictor variable reporting higher average vaccination rates. For the Percent Without Internet and Percent Uninsured, we can see the negative relationship that persists for predicting the vaccination rate of a county, with the lowest level of each of these variables corresponding to higher average vaccination rates.

## 4. Discussion

Recall that the purpose of this study was to determine what demographic, socioeconomic, and political affiliation variables affected the patterns of COVID-19 vaccinations for a single midwestern state over the first 12 months of vaccine rollout. Here we will detail our findings for the variables we selected and discuss how they are related to previous findings. The confidence intervals and *p*-values that are provided in the following discussion come from the month of December, unless otherwise noted. However, since the *p*-values may have changed from month to month, a holistic interpretation of the findings is provided.

### 4.1. Impact of the Predictor Variables on Vaccine Uptake

Our analysis showed that the county level unemployment rate had a consistently strong negative impact on the vaccine uptake in the county (95% CI = [−191,040.36, −59,877.54], *p* < 0.001). This agrees with the study by Guo et al., which found that the unemployment rate of a county was negatively correlated with vaccine uptake in counties with higher predominantly non-Hispanic White populations. Their research posited that BIPOC (Black, Indigenous, and other People of Color) individuals faced higher risks of unemployment during the pandemic. Moreover, if they received the vaccination, they had a higher probability of joining or returning to the workforce [48,49]. It is possible that “the no-cost access to the COVID-19 vaccine for those unemployed and likely without health insurance…may provide a means for those with increased risk of unemployment to secure employment, provided that many employers are starting to require COVID-19 vaccination” [48].

Furthermore, our analysis demonstrated that the county-level uninsured rate had a consistently negative impact on the vaccine uptake in the county (95% CI = [−48,693.43, −7586.94], *p* = 0.008). This agrees with research done by Mollalo and Tatar, who found that the uninsured rate was negatively associated with vaccination rates [10], and Donadio et al. who found that insurance coverage has a strong positive association with vaccination coverage [50]. Mollalo and Tatar believed that one explanation as to why counties in the United States with lower insurance coverage had significantly slower vaccine rollout was because there was a fear among the uninsured population of receiving a bill, despite the vaccine being distributed at no cost [10]. Conversely, Donadio et al. explained that insured individuals find out about their vaccine eligibility status from their primary care provider, and that those without insurance know less about both eligibility for vaccinations and precautions to reduce COVID-19 infection risk [50].

Next, our analysis showed that the county population proportion of those that were of Other Race had a consistent negative impact on the vaccine uptake in the county (95% CI = [−36,198.718, −6439.359], *p* = 0.006). This corroborates findings from other studies like those of Malik et al. [51] and Njoku et al. [52]. Malik et al. found that, because of the disparities in health care for BIPOC individuals, as well as the increased impact of COVID-19, both health-wise and economic-wise, on the BIPOC community, they are less likely to accept a potential COVID-19 vaccine [51]. Furthermore, Njoku et al. found the structural barriers to vaccination and consequent limited vaccine availability for Black and Hispanic populations reduced the vaccine uptake for these populations [52]. The good news is that, from our time series analysis (Table 3 and Table 4), it does appear that this difference may decrease over time as the vaccine became more readily available in all areas and the vaccine earned more trust.

Interestingly, our analysis showed that the proportion of the county population without Internet access was important in predicting the vaccine uptake in the county, though the relationship was very weak (95% CI = [−31,306.54, 5653.49], *p* = 0.171). Recall that the AIC was used for variable selection which, in conjunction with the *p*-value, tells us that Internet access is important for predicting vaccine uptake. However, it was moderately correlated with other variables that were left in the model, such as college education (r = 0.618) and insurance status (r = 0.485), and thus is competing for predictive power. The county level poverty rate was also found to be important for predicting vaccine uptake in a county. At the beginning of the year, this variable was much more impactful (i.e., in May: 95% CI = [−44,862.14, −10,022.66], *p* = 0.002), but by the end of the year its importance had diminished significantly, showing a weak negative impact on the vaccine uptake in the county (95% CI = [−53,422.19, 1510.30], *p* = 0.06). This corroborates the findings by Keegan et al. [53] and Brown et al. [54]. While there are likely many reasons that this sort of relationship could exist, the authors believe that counties with higher rates of uninsured and unemployed individuals, as well as higher proportions of the population that live below the poverty line, will have fewer financial and healthcare resources to both advertise the availability of the vaccine and administer the vaccine. Furthermore, since there is an overlap between unemployed populations and lower-income populations, there may be increased hesitancy to receive a vaccine because these populations have been disproportionately affected by the pandemic, and may be more susceptible to future health problems, even after vaccination [51]. These hypotheses may warrant future testing.

**Table 3:** The Monthly Models Measuring COVID-19 Vaccination for January to June 2021. This table demonstrates that the variables selected by the AIC were consistent for the vaccination patterns in Indiana; after May, the same variables continue to be selected. **Bolded values are significant at the level of *p* < 0.05.**

Variable	January 2021		February 2021		March 2021		April 2021		May 2021		June 2021	
	β (SE)	*p*	β (SE)	*p*	β (SE)	*p*	β (SE)	*p*	β (SE)	*p*	β (SE)	*p*
Intercept	4755.2 (1915.7)	**0.0151**	21,167.1 (8998.2)	**0.02102**	98,626.9 (37,812.1)	**0.010831**	107,785.0 (40,205.2)	**0.00889**	60,267 (7047)	**<0.001**	9017 (6478)	0.167666
% Male			−28,948.4 (17,821.3)	0.10809	−37,228.6 (23,014.5)	0.109634						
% Below Poverty			−16,359.5 (6151.9)	**0.00940**	−27,321.4 (8118.7)	**0.001171**	−29,446.0 (10,116.6)	**0.00465**	−27,442 (8760)	**0.002384**	−19,698 (9969)	0.051491
% College Educated	2390.6 (1132.1)	**0.0378**	3516.7 (2433.0)	0.15210			11,022.7 (3234.9)	**0.00102**	15,729 (2988)	**<0.001**	16,476 (3578)	**<0.001**
% Republican							−42,423.7 (6170.2)	**<0.001**	−47,448 (5254)	**<0.001**		
% Democratic	2287.4 (1433.4)	0.1144	12,718.6 (4532.4)	**0.00625**	33,529.6 (5248.9)	**<0.001**					52,725 (5948)	**<0.001**
% Other Party												
% Non-Hispanic White					−61,787.2 (38,481.5)	0.112246	−57,210.2 (41,317.4)	0.16996				
% Other Race			−11,037.3 (4553.3)	**0.01752**	−88,528.6 (39,481.6)	**0.027677**	−87,647.8 (42,282.8)	**0.04136**	−28,369 (5100)	**<0.001**	−25,159 (5575)	**<0.001**
% Age 18–39	−5296 (3447.1)	0.1283			−16,072.2 (9604.8)	0.098116						
% Age 40–59							38,476.9 (20,369.8)	0.06248	45,087 (18284)	**0.015703**	76,473 (19,955)	**0.000246**
% Age over 60	−7226.2 (4994.3)	0.1517										
RUCC-Nonmetro/Nonrural	208.0 (211.5)	0.3281	840.7 (509.3)	0.10255	1569.7 (647.7)	**0.017604**	1330.1 (710.8)	0.06492				
RUCC-Rural	571.9 (248.2)	**0.0237**	1379.0 (569.1)	**0.01756**	1428.6 (705.6)	**0.046190**	1467.4 (799.1)	0.06998				
% Non-English	−2879.5 (1732.5)	0.1003	−10,141.6 (3598.6)	**0.00604**	−18,735.4 (4609.0)	**0.000111**	−18,898.6 (5850.8)	**0.00179**				
% Unemployment	−11,807 (7201.9)	0.1050			−34,495.4 (21,695.9)	0.115742	−49,767.2 (24,267.1)	0.04352	−55,751 (21,883)	**0.012665**	−85,916 (25,315)	**0.001059**
% Uninsured									−25,464 (6378)	**<0.001**	−18,978 (7513)	**0.013439**
% Non-Internet	−2935.5 (2020.4)	0.1501									−10,338 (6964)	0.141457
% Disability												
Model Adj. R^2^ value	0.301		0.3079		0.5179		0.7241		0.8541		0.8675	
Moran Test *p*-value	0.5528		0.8612		0.6254		0.6002		0.8309		0.7104	
Linear Model AIC	1227.1		1385.88		1425.63		1441.52		1424.46		1438	
SAR Model AIC	1492		1648.7		1690.3		1706.3		1688.1		1702.5	
CAR Model AIC	1492.1		1648.9		1690.3		1706.3		1688.1		1702.5	

**Table 4:** The Monthly Models Measuring COVID-19 Vaccination for July to December 2021. This table demonstrates that the variables selected by the AIC were consistent for the vaccination patterns in Indiana; after May, the same variables continue to be selected. **Bolded values are significant at the level of *p* < 0.05.**

Variable	July 2021		August 2021		September 2021		October 2021		November 2021		December 2021	
	β (SE)	*p*-Value	β (SE)	*p*-Value	β (SE)	*p*-Value	β (SE)	*p*-Value	β (SE)	*p*-Value	β (SE)	*p*-Value
Intercept	8956 (6767)	0.189307	7651 (7108)	0.284849	7709 (7654)	0.316772	8138 (7919)	0.30708	8816 (8030)	0.275409	13,298 (9217)	0.152949
% Male												
% Below Poverty	−18,996 (10,413)	0.071711	−17,785 (10,937)	0.107723	−16,986 (11,778)	0.153011	−16,538 (12,186)	0.17843	−17,139 (12,357)	0.169154	−25,956 (13,804)	0.063665
% College Educated	16,764 (3738)	**<0.001**	15,751 (3926)	**0.000131**	13,921 (4227)	**0.001458**	13,505 (4374)	**0.00274**	14,048 (4435)	**0.002152**	15,774 (5073)	0.002589
% Republican												
% Democratic	53,015 (6212)	**<0.001**	56,044 (6525)	**<0.001**	58,386 (7027)	**<0.001**	57,832 (7270)	**<0.001**	57,507 (7372)	**<0.001**	59,651 (7642)	**<0.001**
% Other Party												
% Non-Hispanic White												
% Other Race	−22,265 (5824)	**0.000254**	−21,706 (6117)	**0.000640**	−22,109 (6587)	**0.001193**	−20,647 (6815)	0.00326	−19,536 (6910)	**0.005888**	−21,319 (7478)	0.005531
% Age 18–39												
% Age 40–59	82,553 (20,844)	**0.000157**	95,296 (21,893)	**<0.001**	106,544 (23,575)	**<0.001**	110,856 (24,393)	**<0.001**	112,017 (24,734)	**<0.001**	111,838 (26,320)	**<0.001**
% Age over 60											−25,567 (17,192)	0.140867
RUCC-Nonmetro/Nonrural												
RUCC-Rural												
% Non-English												
% Unemployment	−90,428 (26,442)	**0.000974**	−100,227 (27,773)	**0.000525**	−114,616 (29,907)	**0.000246**	−115,929 (30,944)	**0.00033**	−118,990 (31,377)	**0.000282**	−125,459 (32,961)	**0.000273**
% Uninsured	−18,884 (7848)	**0.018338**	−19,096 (8243)	**0.022986**	−22,220 (8876)	**0.014263**	−24,148 (9184)	**0.01019**	−25,635 (9312)	**0.007256**	−28,140 (10,330)	**0.007895**
% Non-Internet	−12,175 (7274)	0.097945	−14,090 (7640)	0.068725	−14,150 (8227)	0.089168	−13,509 (8512)	0.11633	−12,884 (8632)	0.139320	−12,826 (9288)	0.171080
% Disability											24,251 (17,986)	0.181308
Model Adj. R^2^ value	0.8652		0.8622		0.8473		0.8385		0.8388		0.8454	
Moran Test *p*-value	0.5528		0.4870		0.3593		0.3678		0.3612		0.5293	
Linear Model AIC	1446.01		1455.05		1468.67		1474.94		1477.5		1484.85	
SAR Model AIC	1702.5		1720.1		1733.7		1740		1742.5		1749.8	
CAR Model AIC	1702.5		1720.1		1733.7		1740		1742.6		1749.8	

Fortunately, there were drivers for counties to experience higher vaccination rates. Unfortunately, these increases were typically driven by factors that we are not in as much control of, such as the proportion of individuals that were college-educated (95% CI = [5679.88, 25,868.77], *p* = 0.003) [51,54]. It is known that these populations had the highest access to the vaccine [55] and, because they are more likely to trust science and medical professionals, they may be better equipped to weigh the risks and benefits of receiving the vaccine [56].

To better understand how the age of a county’s residents affects vaccine uptake, the reader should recall that this was coded as a categorical variable in our dataset. This means that each of the age variables in the table was dependent on the other levels, and the under 18 age group is not shown as it serves as a baseline for the age variable. Thus, we should not look at any of the age rows in Table 3 and Table 4 independently of the others. These results tell us that those in the 40 to 59 age range are more likely to get vaccinated than our baseline group (95% CI = [59,470.74, 164,205.77], *p* < 0.001), and those in the above 60 category (95% CI = [−59,775.09, 8640.46], *p* = 0.141), as well as those in the 18 to 39 age group, have similar vaccination rates as the baseline group, especially as we approached the end of the year.

Finally, our analysis demonstrated that the county-level proportion of Democratic voters had a strong positive impact on the vaccine uptake in the county (95% CI = [44,445.90, 74,856.89], *p* < 0.001). This corroborates findings by Ye [57] and Albrecht [58]. Furthermore, as the year went on, there was a noticeable gap in county cumulative vaccination proportion between Democratic-leaning counties and Republican-leaning counties. It was not surprising to the authors that there was a negative association between vaccine uptake and the proportion of Republican voters within a county. It is well documented that there was mixed messaging from leadership within the party, which led to a distrust of science and medical professionals, and lower vaccination rates [58]. Other studies have found that, at the beginning of the pandemic, Democratic leadership saw COVID-19 as a higher-risk event than their Republican counterparts did [59], leading to the politicization of the management of COVID-19 and the eventual subsequent rollout of COVID-19 vaccines. Therefore, providing citizens with trustworthy information and encouraging leaders that they look up to will be crucial for future successful vaccine rollout campaigns.

Most of the findings presented here are similar to those found in other studies. This shows that national studies that have aggregated data over the entire country are useful at predicting at the state level, at least for Indiana. The authors posit that this may not be the case for all states though. Indiana is what the authors consider an “average” state that does not stand out as being different from the rest of the country. If the study were to be repeated with a different state, such as Texas or California, the authors feel the results may differ. This would be important to research further as more localized vaccine deployment is likely to be more impactful on vaccine uptake.

However, not all variables that were previously found to be impactful may be as significant when considering other competing variables in the model. As was noted above, the proportion of a county having Internet access was not found to be significant, though it was important. The multicollinearity that exists between Internet access and other variables in the model, such as college education and insurance status, may need to be researched further to determine how these specific variables interact with each other and their role in predicting vaccine uptake.

One particularly interesting finding of this study was the impact of different variables on vaccine uptake over time. We can see from the graphs in Figure 5 and from Table 3 and Table 4 that some of the variables that we identified as playing a role in vaccine uptake changed their level of significance over time. For example, we saw this with the county poverty rates. This indicates that it is important to spearhead initiatives early on that prevent these variables from becoming problematic several months after the vaccine has been rolled out. We continue the paper by providing some suggestions for future vaccine rollout that could help with these initiatives.

### 4.2. Potential Methods to Improve Vaccine Uptake in the Future

Vaccine rates could be impacted for the future in a variety of ways. For example, targeted messaging is a strategy that provides culturally relevant advertising campaigns to reach groups that are less likely to get a vaccine, like Republican-voting individuals [30], communities that have mistrust in the healthcare system [55], and uninsured populations [11,58]. For example, it may be more effective for individuals who have a mistrust in government to disseminate information about vaccination through companies or primary care physicians. Another means would be to give information about side effects or possible allergic reactions individuals might have to the vaccine. Also, health departments could partner with organizations at the local and community levels to promote vaccine efficacy and uptake to demonstrate that it is safe to receive and improves the community at the local level. Furthermore, the government could mandate employers to provide a normal hourly salary to any employee that misses work within 24 h of receiving the vaccine due to its side effects.

It is also crucial for health departments to increase access to rural, under-resourced, and underserved areas. During the pandemic, vaccine distribution was mainly concentrated in metropolitan and suburban areas [60]. Even though rural populations are less likely to spread the disease because they are less densely populated, they are less likely to have insurance than suburban and metropolitan counties. If individuals from these rural populations get sick, then they may have a harder time paying for their medical care and have a harder time finding a medical provider due to distance.

Our study also found that, at the beginning of the vaccine rollout, there was a negative association between the proportion of non-English speakers and the county vaccine uptake. One way to increase access to these populations is to provide documentation and vaccine registration in multiple languages to remove another structural barrier to vaccination. Registration itself could also be simplified so that it would not require nonessential documentation, like proof of US citizenship, which could deter individuals from immigrant communities, especially undocumented persons, from receiving the COVID-19 vaccine [61].

At the same time, outreach is needed for populations that are of lower socioeconomic status. One means of increasing vaccination access in these underserved health areas may be through mobile vaccination clinics. These vaccination clinics could be at hours of the day that are convenient for those that are working full-time to receive it outside of work hours. Furthermore, these clinics should be appointment-free, or through registration by telephone or in-person, to increase access for those without reliable Internet access [61,62].

Finally, it is crucial that each of these things happen early in vaccine rollout. Our analysis showed that many of these variables did not have a notable effect on vaccine uptake early in the vaccine rollout process. However, the effects seemed to compound as the year went on. The authors believe that early targeting for each of these issues could significantly increase vaccine update and reduce disparities between groups.

### 4.3. Study Limitations

Our study has some notable limitations. First, due to the cross-sectional and ecological nature of the data, we were unable to draw causal inferences or downscale inference to smaller geographic units. Future analyses may employ causal inference methods to make more informed inferences. Additionally, while obtaining data on an individual level may never be feasible for HIPAA reasons, there is an abundance of county-level data that could be used for studies like this one. Previous studies suggest that there can be significant heterogeneity in attitudes toward COVID-19 infections and vaccinations within a given county, meaning that the current scale may be reasonable for modeling future vaccination attempts.

Second, we operationalized county-level political affiliation using the proportion of Democrat, Republican, and Other voters in the 2020 U.S. Presidential Election, which is an imperfect proxy for political ideology or community engagement. However, other studies have utilized a similar methodology for approximating political affiliation as a variable.

Third, while we did attempt to account for patterns in the month-to-month COVID-19 vaccines, a formal time series analysis was not feasible. This was because the time-lagged vaccine per 100,000 count value was much larger than the other predictor variables, causing it to overpower the model, which did not allow us to see the effects other variables had on vaccine uptake.

Finally, although the findings may not generalize to all other states, we have shown that findings can generalize from the country level to the state level, particularly Indiana, which suggests that they may do so to others as well.

## 5. Conclusions

Despite these limitations, this study offers an important implication for future pandemics and other public health emergencies. We showed that linking county-level demographic, socioeconomic, and political affiliation characteristics with COVID-19 vaccination patterns revealed associations between vaccination patterns and poverty rate, unemployment rate, uninsured rate, county population proportion without access to the Internet, proportion of the population of Other Race, college-educated individuals, population ages 40–59, and political affiliation, confirming findings of several large-scale studies for a midwestern state. Through this analysis of Indiana, we demonstrated that models measuring vaccinations for the entire country masked other potential relationships seen at the micro level over time. These results suggest that in future public health disasters, tailored public health messaging and reallocation of resources to areas with less access to healthcare are essential for a successful public health response.

While COVID-19 seems to be a thing of the past for many of us, the information that was gained on vaccine adoption will be helpful when considering the rollout of future vaccines. By learning factors that were helpful at increasing vaccine adoption, as well as those that hindered it, we may be able to better inform future policies about where vaccines should be sent, messaging that should be made, and how to distribute vaccines equitably.

## Figures and Tables

**Table 1:** Rural–Urban Commuting Codes. Data Source: [46].

RUCC	Original Description	Reclassification
1	Metro: Counties in metro areas of 1 million population or more	Metro
2	Metro: Counties in metro areas of 250,000 to 1 million population	Metro
3	Metro: Counties in metro areas of fewer than 250,000 population	Metro
4	Non-Metro: Urban population of 20,000 or more, adjacent to a metro area	Nonmetro/Nonrural
5	Non-Metro: Urban population of 20,000 or more, not adjacent to a metro area	Rural
6	Non-Metro: Urban population of 5000 to 20,000, adjacent to a metro area	Nonmetro/Nonrural
7	Non-Metro: Urban population of 5000 to 20,000, not adjacent to a metro area	Rural
8	Rural: Urban population of fewer than 5000, adjacent to a metro area	Rural
9	Rural: Urban population of fewer than 5000, not adjacent to a metro area	Rural

**Table 2:** Summary statistics for the demographic, socioeconomic, and political affiliation characteristics for the 92 counties in the state of Indiana.

Variable	Mean	SD
COVID Vaccinations per 100,000	46,516/100,000	7686
% Non-Hispanic White	0.8975	0.0794
% Other Race	0.0827	0.0750
18–39	0.2663	0.0376
40–59	0.2604	0.0189
Above 60	0.2448	0.0285
% College Educated	0.2817	0.1226
% Republican	0.6796	0.0962
% Democratic	0.2880	0.0965
% Other Party	0.0324	0.0124
% Non-English Speakers	0.0557	0.0590
% Unemployed	0.0425	0.0128
% Uninsured	0.0817	0.0479
% Non-Internet	0.1890	0.0545
% Disability	0.1533	0.0290
% Below Poverty	0.1218	0.0386

## Data Availability

The original data and code presented in the study are openly available in GitHub at https://doi.org/10.5281/zenodo.11187671 (accessed on 27 June 2024).
